# Prognostic and Immunological Significance of the Molecular Subtypes and Risk Signatures Based on Cuproptosis in Hepatocellular Carcinoma

**DOI:** 10.1155/2023/3951940

**Published:** 2023-04-20

**Authors:** Xiaolong Tang, Xiangqing Ren, Tian Huang, Yandong Miao, Wuhua Ha, Zheng Li, Lixia Yang, Denghai Mi

**Affiliations:** ^1^The First Clinical Medical College, Lanzhou University, Lanzhou City, Gansu Province, China; ^2^The Second Department of Gastrointestinal Surgery, Affiliated Hospital of North Sichuan Medical College, Nanchong City, Sichuan Province, China; ^3^The Second Department of Medical Oncology, Yantai Affiliated Hospital of Binzhou Medical University, The 2nd Medical College of Binzhou Medical University, Yantai City, Shandong Province, China; ^4^Institute of Modern Physics, Chinese Academy of Sciences, Lanzhou City, Gansu Province, China; ^5^Gansu Academy of Traditional Chinese Medicine, Lanzhou City, Gansu Province, China

## Abstract

**Background:**

Hepatocellular carcinoma (HCC) remains a challenging medical problem. Cuproptosis is a novel form of cell death that plays a crucial role in tumorigenesis, angiogenesis, and metastasis. However, it remains unclear whether cuproptosis-related genes (CRGs) influence the outcomes and immune microenvironment of HCC patients.

**Method:**

From The Cancer Genome Atlas (TCGA) and International Cancer Genome Consortium (ICGC) databases, we obtained the mRNA expression file and related clinical information of HCC patients. We selected 19 CRGs as candidate genes for this study according to previous literature. We performed a differential expression analysis of the 19 CRGs between malignant and precancerous tissue. Based on the 19 CRGs, we enrolled cluster analysis to identify cuproptosis-related subtypes of HCC patients. A prognostic risk signature was created utilizing univariate Cox regression and least absolute shrinkage and selection operator (LASSO) regression analyses. We employed independent and stratification survival analyses to investigate the predictive value of this model. The functional enrichment features, mutation signatures, immune profile, and response to immunotherapy of HCC patients were also investigated according to the two molecular subtypes and the prognostic signature.

**Results:**

We found that 17 CRGs significantly differed in HCC versus normal samples. Cluster analysis showed two distinct molecular subtypes of cuproptosis. Cluster 1 is preferentially related to poor prognosis, high activity of immune response signaling, high mutant frequency of *TP53*, and distinct immune cell infiltration versus cluster 2. Through univariate and LASSO Cox regression analyses, we created a cuproptosis-related prognostic risk signature containing *LIPT1*, *DLAT*, *MTF1*, *GLS*, and *CDKN2A*. High-risk HCC patients were shown to have a worse prognosis. The risk signature was proved to be an independent predictor of prognosis in both the TCGA and ICGC datasets, according to multivariate analysis. The signature also performed well in different stratification of clinical features. The immune cells, which included regulatory T cells (Treg), B cells, macrophages, mast cells, NK cells, and aDCs, as well as immune functions containing cytolytic activity, MHC class I, and type II IFN response, were remarkably distinct between the high-risk and low-risk groups. The tumor immune dysfunction and exclusion (TIDE) score suggested that high-risk patients had a higher response rate to immune checkpoint inhibitors than low-risk patients.

**Conclusion:**

This research discovered the potential prognostic and immunological significance of cuproptosis in HCC, improved the understanding of cuproptosis, and may deliver new directions for developing more efficacious therapeutic techniques for HCC patients.

## 1. Introduction

Primary liver cancer remains a serious threat to global public health in 2020, with over 906,000 new cases and 830,000 fatalities [1]. Most liver cancer cases involve hepatocellular carcinoma (HCC), which accounts for about 90%. Chronic hepatitis B or C virus infection, alcohol abuse, and metabolic syndrome induced by obesity and diabetes are the key risk elements for the occurrence of HCC [2]. Early-stage HCC is remediable through surgery or ablation. Nevertheless, there are very few medicinal choices available for advanced-stage HCC patients due to strong and broad resistance to cytotoxic chemotherapy [3]. Sorafenib, a multityrosine kinase inhibitor (mTKI) with antiangiogenic and antiproliferative properties, has been the regular first-line treatment for advanced HCC for more than a decade [4, 5]. Nevertheless, it is estimated that only a small ratio of HCC patients responds to sorafenib. Up to now, significant efforts have been dedicated to enhancing the medicinal condition of advanced-stage HCC patients in the past few years, with the approval of four agents: lenvatinib [6] as front-line treatment, ramucirumab [7], cabozantinib [8], and regorafenib [9] as second-line treatment. By utilizing antiprogrammed death protein 1 (*PD-1*) antibodies, immune checkpoint blockade (ICB) has been licensed to treat HCC in second-line [10, 11]. Despite a marked increase in the choice of systemic therapies, there has been a modest improvement in patient outcomes. Therefore, there is a pressing demand for new molecular biomarkers for HCC patients in order to guide more personalized treatment decisions.

Copper is an essential nutrient for the human body. Meanwhile, copper can cause cell death via cytotoxicity, which is driven by improved mitochondrial-dependent energy metabolism and increased reactive oxygen species (ROS). The phenomenon of cell death because of copper is termed “cuproptosis” [12]. Specifically, cuproptosis is caused by copper in combination with lipidated components of the tricarboxylic acid cycle (TCA). Eventually, proteotoxic pressure and cell death occur as a lack of iron-sulfur cluster proteins and a result of lipoylated protein aggregation. Cuproptosis was classified as a new kind of regulated cell death distinct from the recognized cell death pathway, such as apoptosis, necrosis, autophagy, ferroptosis, and pyroptosis [12]. The process of cell death is inextricably linked to tumor development and the immune microenvironment [13]. The potential role of cuproptosis in clinical outcomes, as well as its immune characterization, warrants further study. As for cancer treatment, ionophores for copper supplementation are the primary current therapeutic approach based on cuproptosis, including disulfiram (DSF) and elesclomol [14–16]. In Wilson's disease, due to *ATP7B* deletion, a progressive hepatic copper overload may happen in the hepatocytes and lead to liver failure [17]. The improved incidence of HCC in patients with Wilson's disease provides evidence that aberrant copper homeostasis may contribute to HCC development through an unknown mechanism [18]. In both *in vitro* [19] and *in vivo* [20], copper excess caused cell death in hepatocytes through the intrinsic pathway. Nevertheless, cuproptosis's contribution to the tumorigenesis and development of HCC has not yet been fully understood.

In our study, we identified a substantial variance in the expression level of cuproptosis-related genes (CRGs) between malignant and precancerous tissue, which may reveal the close relationship between CRGs and HCC development. Next, we performed the consensus cluster analysis and identified two cuproptosis-associated clusters, which were significantly likened to patient survival and immune characterization. We then explored the prognostic value of CRGs for the outcomes of HCC patients and built a prognostic risk model containing five CRGs to predict prognosis, somatic mutation signature, immune microenvironment, and response to immunotherapy in HCC. According to multiple datasets, including The Cancer Genome Atlas (TCGA) and International Cancer Genome Consortium (ICGC), this risk model performed high accuracy in evaluating HCC prognoses. These findings can contribute to further insight into the importance of CRGs in HCC development and support further clinical development of cuproptosis for HCC.

## 2. Materials and Methods

### 2.1. Data Resources and Preprocessing

The TCGA (https://portal.gdc.cancer.gov/) provided the somatic mutation information, mRNA expression profile, and matched clinical data for liver hepatocellular carcinoma (LIHC) cases. Through the ICGC (https://dcc.icgc.org/), the project (code: LIRI_JP) on liver cancer was downloaded. R (version 4.2.0) software was enrolled to collate and annotate the somatic mutation and RNA-sequencing data. The TCGA database contained 50 normal tissues and 374 tumor tissues, and the ICGC database contained 202 normal tissues and 243 normal tissues. Then, the mRNA expression files were standardized with fragments per kilobase per million mapped reads (FPKM). To scale data among different databases, we adopted the “scale” function in the “limma” R package [21].

### 2.2. Exploration of the Differentially Expressed CRGs in HCC

The cuproptosis-related differentially expressed genes (DEGs) were detected between malignant and precancerous tissue of HCC patients in the TCGA with the “limma” R package. *p* values <0.05 were regarded as the cutoff values for identifying DEGs. Through the “heatmap” R package [22], we generated a heatmap of cuproptosis-related DEG expression levels between HCC and normal tissue. To better know the connections among CRGs, we examined the relationship between CRGs through Pearson's correlation analysis. The online tool STRING [23] was taken advantage to conduct protein-protein interactions (PPI) network, and the Cytoscape tool was enrolled to picture the network [24].

### 2.3. Consensus Clustering Analysis

To further investigate the biological features of CRGs in HCC, with the “ConsensusClusterPlus” R package [25], the patients in TCGA were classified into two clusters according to the 19 CRGs.

### 2.4. Functional Enrichment Analysis

To discover the gene functions and biological pathways of the CRGs, we operated Gene Ontology (GO) and Kyoto Encyclopedia of Genes and Genomes (KEGG) pathway enrichment analyses of CRGs utilizing the R packages “limma” and “clusterProfiler” [26]. Next, we conducted Gene Set Enrichment Analysis (GSEA) by employing the GSEA tool [27] against gene sets from the MSigDB. To further assess the biological function differences between the groups, GSVA enrichment analysis was performed based on the “GSVA” R package [28].

### 2.5. Somatic Mutational Hotspot Analysis

Through the GDC data portal at TCGA, we got the somatic mutation data with the Mutation Annotation Format (MAF) for HCC patients. The “Maftools” R package [29] in R software was utilized for summarization and visualization of the mutated genes.

### 2.6. Construction and Validation of a Novel Prognostic Model Based on CRGs

According to univariate Cox analysis, we screened out survival-associated genes, and with the least absolute shrinkage and selection operator (LASSO) Cox regression, we formed a risk signature through the “glmnet” and “survival” R packages [30] in the TCGA. The risk score was computed as follows:

(1) $$\begin{array}{c} \text{risk score}=\overset{n}{\underset{j=1}{\sum }}\text{C}\text{oef }j*\text{xj}. \end{array}$$

xj on behalf of the expression levels of every prognostic CRG and Coef on behalf of the coefficient. According to the median score, HCC patients were categorized into low- and high-risk groups. Kaplan-Meier survival curves were utilized to compare the two groups' overall survival (OS) and progression-free survival (PFS). The “timeROC” R package was utilized to calculate the time-dependent receiver operating characteristic (ROC) curve according to the signature's sensitivity and specificity [31]. We employed univariate and multivariate Cox regression analysis to test the risk score's independent prognostic value. Chi-square examinations were utilized to measure the association between risk levels and clinical characteristics. Next, the ICGC database was used to verify the risk score's predictive ability. The same formula used for TCGA patients was enrolled to estimate the risk scores of ICGC patients.

### 2.7. Kaplan–Meier Survival Curve Analysis

Kaplan-Meier survival curves were adopted for survival analysis with Mantel-Wilcox tests. We conducted a survival analysis of HCC cases in the TCGA database based on gene clusters, risk groups, and clinical features stratification, while HCC patients in the ICGC were analyzed according to risk groups.

### 2.8. Construction of Prognostic Nomograms

Through the R package “rms” [32], we constructed a nomogram and corresponding calibration map through the risk score and other important clinical traits. The area under the ROC curve (AUC) was utilized to measure the diagnostic power of the nomogram. Univariate and multivariate Cox regressions were employed to assess whether the nomogram was an independent predictor.

### 2.9. Tumor-Infiltrating Immune Cells Analysis

To comprehensively assess the composition of tumor-infiltrating immune cells, we employed several methods, including TIMER, CIBERSORT, CIBERSORT-ABS, QUANTISEQ, MCPcounter, XCELL, EPIC, and ssGSEA. Correlation analysis was used to examine the relationship between immune cell infiltration and risk level. Furthermore, eight critical genes involved in immune checkpoint blockade therapy were extracted from each case and compared between different groups, including *TIGIT*, *PD-L2*, *PD-L1*, *PD-1*, *LAG3*, *SIGLEC15*, *TIM-3*, and *CTLA-4*.

### 2.10. Immunotherapy Response Predictions

Tumor immune dysfunction and exclusion (TIDE) [33] was enrolled to figure out how probable it was that HCC patients' responses to ICB.

## 3. Results

### 3.1. Exploration of Differentially Expressed CRGs in HCC

**Figure 1:**
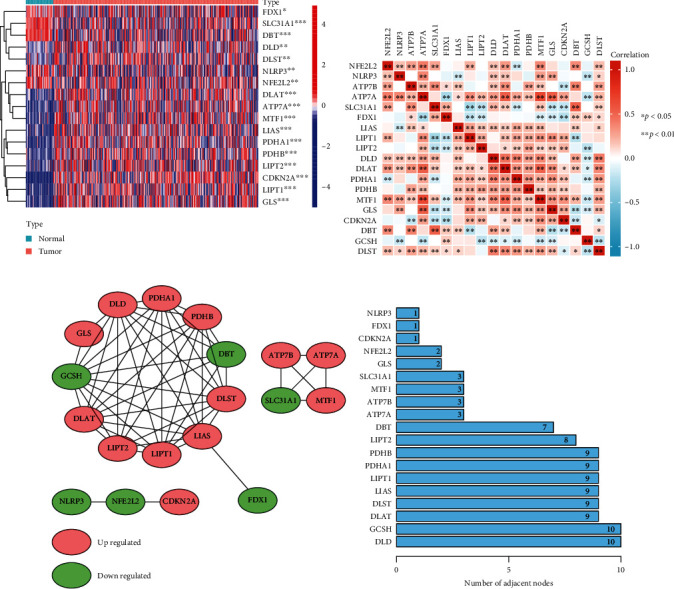
Identification of cuproptosis-related DEGs and exploration of the relationship between each CRG in HCC based on the TCGA database. (a) Cuproptosis-related DEGs expression patterns between HCC and normal tissue. The color legend represents the log2 (FPKM) value. (b) Pearson's correlation analysis of each CRG based on the HCC samples. (c) PPI network plot displayed the relationship between each CRG. Red and green nodes indicate up and downregulated genes, respectively. (d) The number of adjacent nodes between each CRG in the PPI network. ^∗^*p* < 0.05, ^∗∗^*p* < 0.01, ^∗∗∗^*p* < 0.001.

We carefully selected a gene set of 19 genes (*ATP7B*, *ATP7A*, *DLD*, *DLAT*, *DLST*, *SLC31A1*, *FDX1*, *LIPT1*, *LIAS*, *LIPT2*, *PDHA1*, *NFE2L2*, *NLRP3*, *GLS*, *MTF1*, *CDKN2A*, *GCSH*, *DBT*, and *PDHB*) which function closely with cuproptosis. The screening criteria of the 19 CRGs were based on the core literature reported by Tsvetkov et al., who first defined the cuproptosis [12]. In the TCGA, compared to normal tissues, 17 genes were differentially expressed in HCC, including *ATP7A*, *DLD*, *DLAT*, *DLST*, *SLC31A1*, *FDX1*, *LIPT1*, *LIAS*, *LIPT2*, *PDHA1*, *NFE2L2*, *NLRP3*, *GLS*, *MTF1*, *CDKN2A*, *DBT*, and *PDHB* (Figure 1(a)). Based on the HCC samples, the relationship between CRGs was then revealed using Pearson's correlation analysis (Figure 1(b)). Next, the PPI network was formed by the web tool STRING and pictured through the Cytoscape program to further reveal the potential connection between the related proteins (Figure 1(c)). In the PPI network, we counted the number of adjacent nodes (Figure 1(d)). We discovered a strong correlation between each CRG in HCC tissues, suggesting that these CRGs may act as a whole and perform a common function of cuproptosis together. These findings demonstrated that CRGs' expression patterns between HCC and normal tissues are remarkably different, indicating that CRGs may perform a significant function in the tumorigenesis and development of HCC.

### 3.2. Consensus Clustering Identified Two Cuproptosis-Associated Subtypes and Survival Analysis

**Figure 2:**
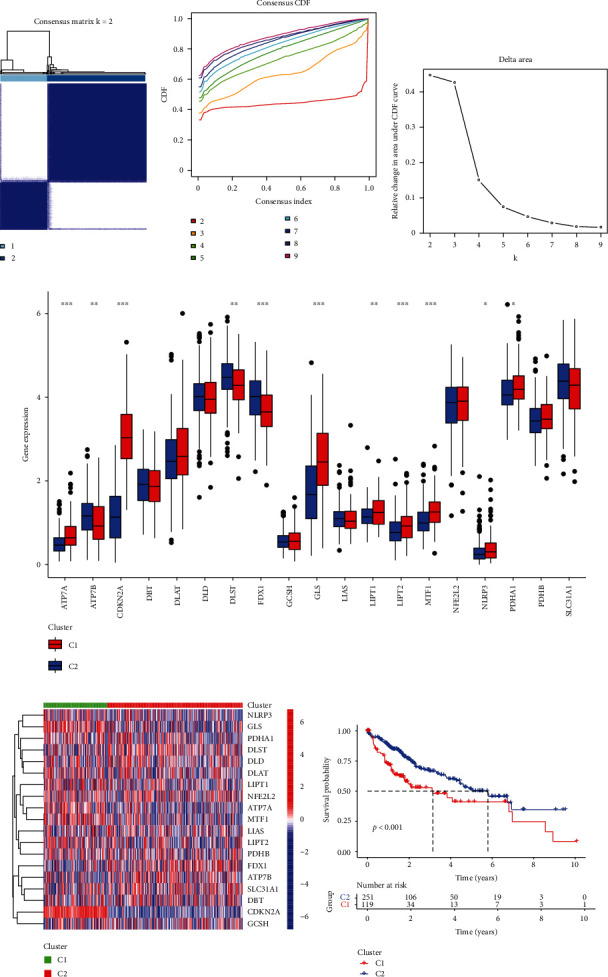
Consensus clustering of cuproptosis-associated subtypes and survival analysis in the TCGA. (a) Heatmap represented the consensus clustering solution (*k* = 2) for 19 CRGs among 502 HCC samples. (b, c) The consensus clustering delta area showed the cumulative distribution function area for *k* = 2 to 9. (d) Boxplots represented gene expression profiles for 19 genes in the two clusters. (e) An expression heatmap showed 19 genes grouped into two clusters. The color legend represents the log2 (FPKM) value. Red highlighted the high expression, and blue highlighted the low expression. (f) Kaplan–Meier curves of OS in different clusters. ^∗^*p* < 0.05, ^∗∗^*p* < 0.01, ^∗∗∗^*p* < 0.001.

To reveal the relationship between cuproptosis subtypes and HCC patients' clinical outcomes, we used 19 CRGs to cluster HCC patients in the TCGA database. Through K-means cluster analysis, HCC patients were clustered into two subgroups according to the 19 CRGs with similar expression patterns (Figures 2(a)–2(c)). The gene expression data of 19 CRGs in two clusters showed that a high expression level of *ATP7A*, *CDK2A*, *GLS*, *LIPT1*, *LIPT2*, *MTF1*, *NLRP3*, and *PDHA1* was found in cluster 1, while cluster 2 showed high expression levels of *ATP7B*, *DLST*, and *FDX1* (Figures 2(d), 2(e)). The Kaplan-Meier analysis of survival discovered that the clusters linked with cuproptosis had distinct clinical outcomes. Patients in cluster 1 had poorer clinical results, whereas those in cluster 2 had a more favorable prognosis (Figure 2(f)). These results revealed that there might be a relationship between cuproptosis-associated subtypes and HCC clinical outcomes.

### 3.3. Functional Enrichment Analysis Based on Clustering

**Figure 3:**
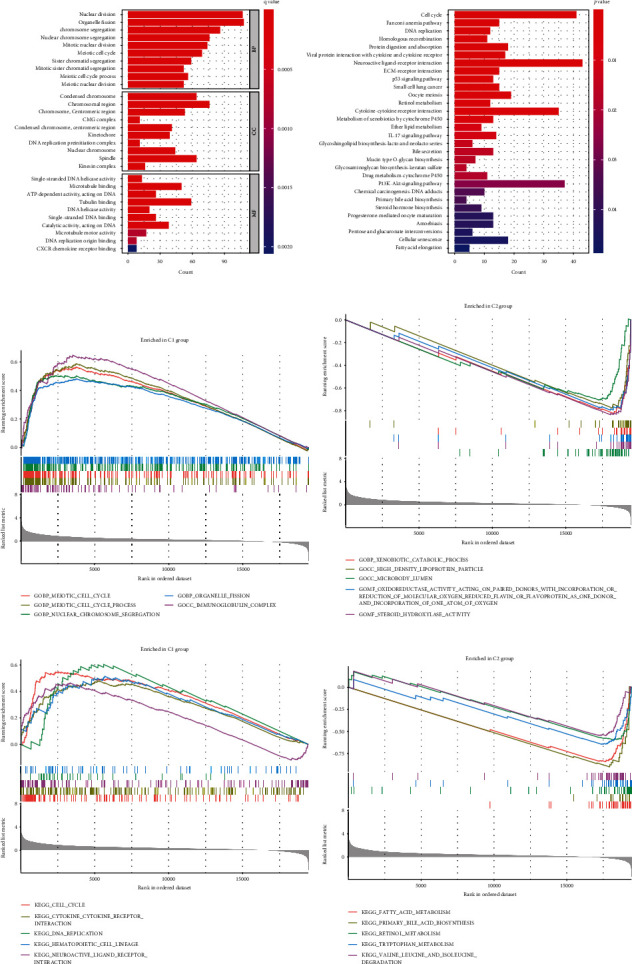
Results of functional enrichment analysis. (a) A list of the top 10 enriched GO terms. Topics contained biological processes (BP), cellular components (CC), and molecular functions (MF). (b) The top 30 most significant enriched KEGG pathways. (c) The top 5 GSEA-GO enrichment in cluster 1. (d) The top 5 GSEA-GO enrichment in cluster 2. (e) The top 5 GSEA-KEGG enrichment in cluster 1. (f) The top 5 GSEA-KEGG enrichment in cluster 2.

GO, KEGG, and GSEA analyses were conducted on the DEGs between two clusters with cut-off criteria of *p* value <0.05 and |log2FC| ≥ 1 in order to study the biological function variations of each cluster. We presented the top 10 GO terms, 30 significant enriched KEGG pathways, and the top 5 normalized enrichment scores terms of GSEA. Among GO terms, nuclear division, mitotic nuclear division, condensed chromosomes, and single-stranded DNA helicase activity were significantly enriched (Figure 3(a)). On the KEGG pathway list, DNA replication, cell cycle, p53, and IL-17 signaling pathways are significantly enriched (Figure 3(b)). According to GESA, two clusters had differentially enriched gene sets. Based on GSEA, GO terms in cluster 1 are predominantly associated with cell cycle, nuclear chromosome segregation, organelle fission, and immunoglobulin complex (Figure 3(c)). GO terms in cluster 2 were enriched in xenobiotic catabolic processes, high-density lipoprotein particles, and microbody lumens (Figure 3(d)). The KEGG pathways in cluster 1 were predominantly related to DNA replication, cell cycle, and cytokine-cytokine receptor interaction (Figure 3(e)). As for cluster 2, it was enriched in fatty acid metabolism, bile acid production, and retinol production (Figure 3(f)). According to these results, the two clusters differ in biological function, and the differences mainly focus on cell cycle, cell death, and immune-related functions.

### 3.4. Somatic Mutations and Immune Landscape of Cuproptosis-Related Clusters

**Figure 4:**
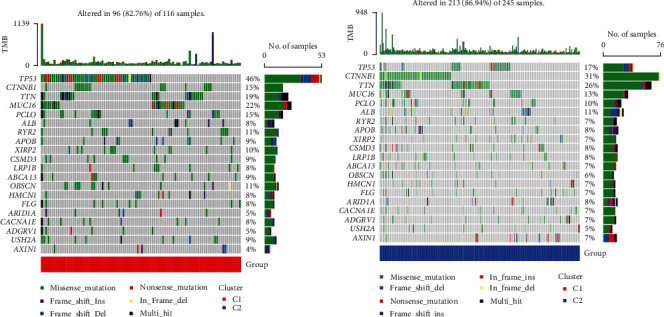
The somatic mutations landscape of two cuproptosis-related clusters. The top ten mutated genes in cluster 1 (a) and cluster 2 (b) were visualized using a waterfall plot.

**Figure 5:**
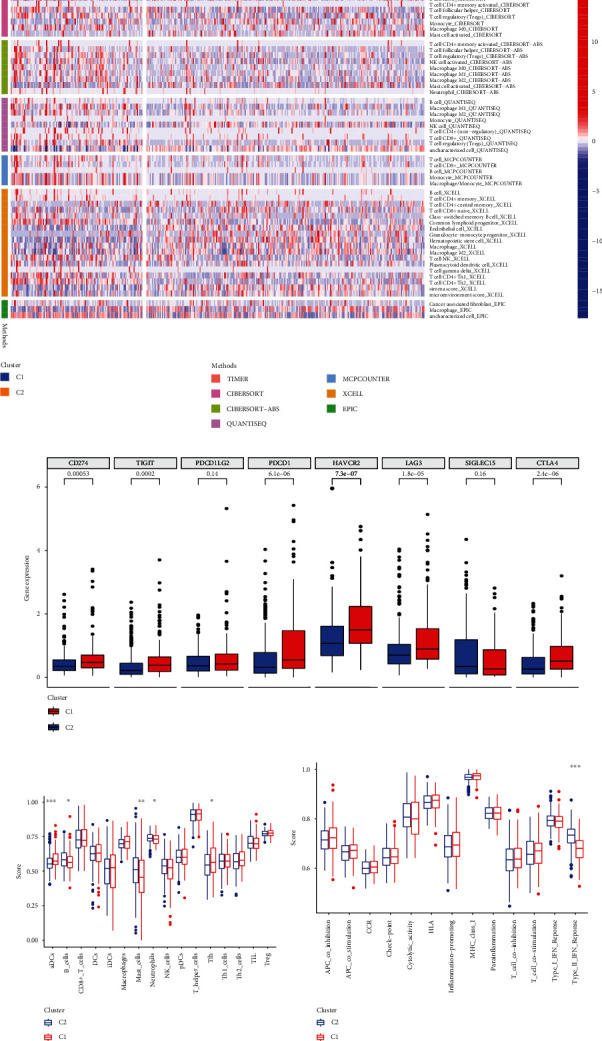
The immune landscape of two cuproptosis-related clusters in HCC. (a) The immune infiltration heatmap between the two clusters using TIMER, CIBERSORT, CIBERSORT-ABS, QUANTISEQ, MCPcounter, XCELL, and EPIC algorithms. (b) The gene expression levels of immune checkpoints for the two clusters. (c) The ssGSEA for examining subpopulation associations in immune cells. (d) The ssGSEA for examining subpopulation associations in immune functions. ^∗^*p* < 0.05, ^∗∗^*p* < 0.01, ^∗∗∗^*p* < 0.001.

In addition, we investigated the mutation profile of cuproptosis-related clusters in HCC patients. *TP53*, *CDKN2A*, *TTN*, *MUC16*, and *FAT1* were the most abundant mutant genes. The relative mutation frequencies differ between the two clusters. A high frequency of *MUC16* and *TP53* mutations was observed in cluster 1, with 46% and 22% of the total, respectively (Figure 4(a)). In cluster 2, *CTNNB1* and *TTN* were the most frequently mutated genes, with 31% and 26% of the total, respectively (Figure 4(b)). The tumor immune microenvironment in the two clusters needs to be investigated further, then TIMER, CIBERSORT, CIBERSORT-ABS, QUANTISEQ, MCPcounter, XCELL, and EPIC algorithms were used to visualize the immune cell infiltration situation (Figure 5(a)). Immune infiltration of various immune cells differed significantly between the two clusters (Supplementary Table 1). We further investigated immune checkpoint gene expression levels in the eight important immune checkpoints across the two clusters. The expression of *CD274*, *TIGIT*, *PDCD1*, *HAVCR2*, *LAG3*, and *CTLA4* was substantially different between the two clusters of HCC patients (Figure 5(b)). Based on ssGSEA analysis, we analyzed immune cell subpopulations and their related functions. The results revealed that aDCs, B cells, mast cells, neutrophils, Tfh, and type II IFN response significantly differed between the two clusters (Figures 5(c) and 5(d)). According to these findings, there were significant differences between the two cuproptosis-related clusters of HCC in terms of somatic mutations and immune landscape.

### 3.5. Construction of the Cuproptosis-Related Prediction Model in HCC

**Figure 6:**
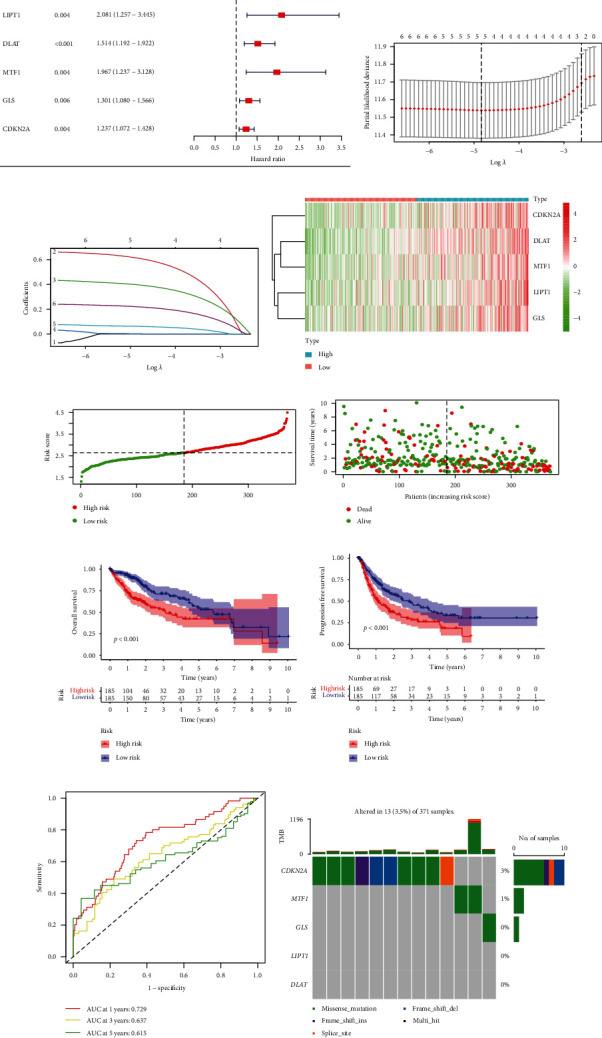
Formation of the risk score signature utilizing five CRGs in the TCGA. (a) Univariate Cox regression analysis selected six CRGs. (b, c) Detection of five prognostic CRGs using the LASSO Cox regression analysis. (d) Heatmaps of the five prognostic CRGs according to the distribution of risk scores. The color legend represents the log2 (FPKM) value. (e) The distribution of risk scores. (f) Patients' survival status according to the distribution of risk scores. (g) Kaplan-Meier survival analysis compared the OS between the high-risk and low-risk groups. (h) Kaplan-Meier survival analysis compared the PFS between the high-risk and low-risk groups. (i) The ROC curves for 1, 3, and 5 years of the risk model. (j) Mutation landscape of the five CRGs of the risk model.

To detect the key genes in cuproptosis and explore the possibility of clinical application of cuproptosis-related phenotype, we developed a prognostic model by differentially expressed CRGs in the TCGA database. Through the univariate Cox analysis, we found six CRGs were significantly associated with OS (Figure 6(a)). Then, five genes were selected in the prognostic model with LASSO Cox regression (Figures 6(b) and 6(c)). The risk-score model is formed as the following algorithm: risk score = (0.6125)^∗^*LIPT*1 + (0.3970)^∗^*DLAT* + (0.0013)^∗^*MTF*1 + (0.0619)^∗^*GLS* + (0.2198)^∗^*CDKN*2*A*. Additionally, according to the distribution of risk scores and survival time, we found higher risk levels were linked to shorter survival times (Figures 6(d)–6(f)). Utilizing Kaplan-Meier analysis, we further assessed the prognostic relevance of this risk profile. A negative correlation was found between risk scores with OS and PFS (Figures 6(g) and 6(h)). Using the ROC curve, we assessed the predictive role of risk score by computing AUC, which was 0.729, 0.637, and 0.615 for the 1-, 3-, and 5-year survival (Figure 6(i)). In addition, we explored the somatic mutation condition of the five model genes. *CDKN2A* is mutated in 3 percent of HCC patients, *MTF1* is mutated in 1 percent of HCC patients, and fewer mutations are found in *GLS*, *LIPT1*, and *DLAT*. The most abundant mutation type is the missense mutation (Figure 6(j)).

### 3.6. Exploration of the Independent Prognostic Value and Clinical Feature of the Risk Score in HCC

**Figure 7:**
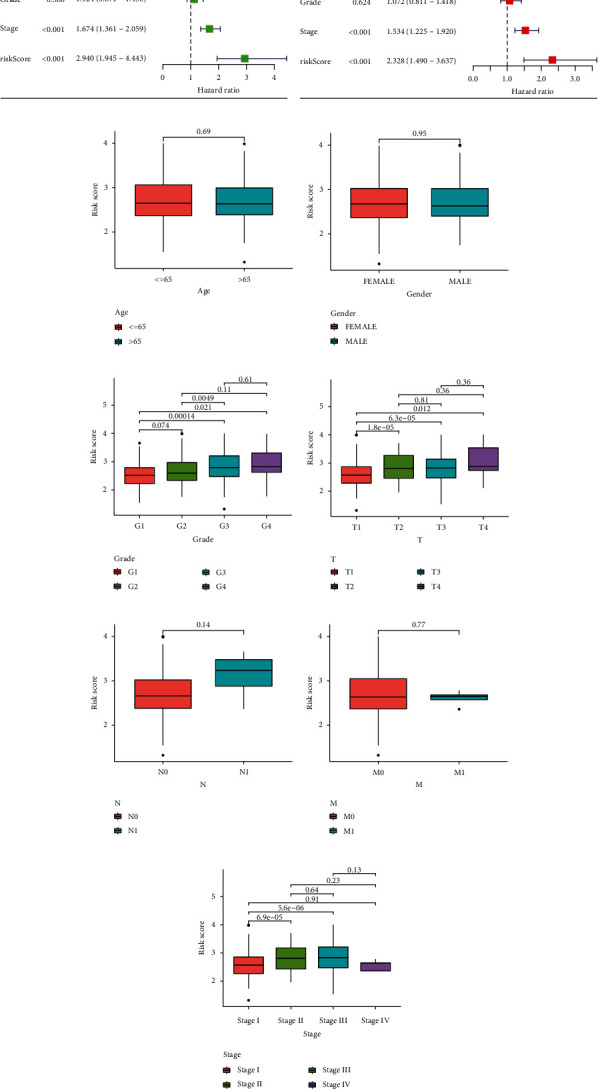
Exploration of the independent prognostic value and clinical feature of the risk score in HCC. (a, b) Through univariate and multivariate Cox regression analysis, the risk score was found to be an independent prognostic element for HCC patients. (c–i) The relationship between the risk score and different clinical parameters of HCC.

In the TCGA, we conducted both univariate and multivariate Cox regression analyses to explore whether risk score and other clinical traits were independent prognostic factors. With five parameters (age, gender, stage, grade, and risk score), the risk score obtained by our formula served as an independent predictor of survival for HCC patients (*p* < 0.01, Figures 7(a) and 7(b)). Meanwhile, it was discovered that the risk score was substantially associated with the tumor stage and grade (Supplementary Table 2). Besides, we compared the risk score across different clinical traits. Interestingly, according to the risk score, we found differences were significant between the T1 stage versus T2, T3, and T4 stage (*p* < 0.05, Figure 7(f)) and tumor stage I versus stage II, and stage III (*p* < 0.01, Figure 7(i)). The other clinical characteristics were also compared separately (Figures 7(c)–7(i)). The high-risk group patients had advanced T stage and tumor stage compared with low-risk group patients. These results indicated that the risk model built with these five genes is capable of accurately predicting the prognosis of HCC.

### 3.7. Implication of Risk Score on the Prognosis of HCC Patients in Different Clinical Parameters Stratification

**Figure 8:**
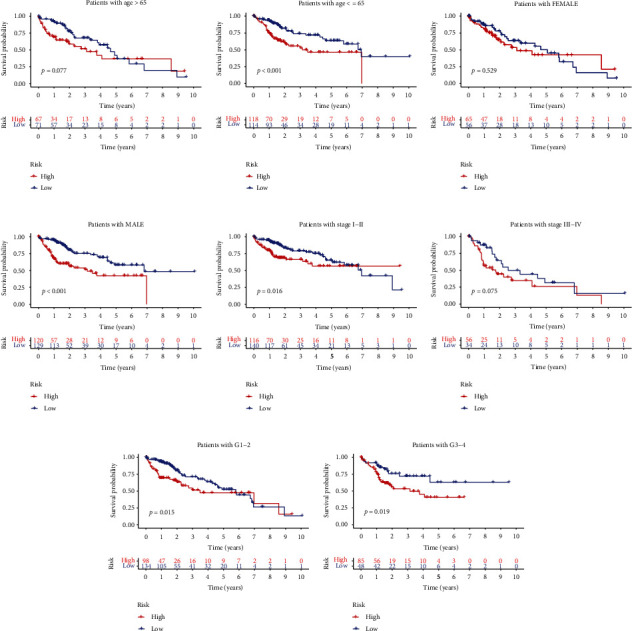
Kaplan-Meier analysis of the risk score in different stratifications according to clinicopathological characteristics. (a–h) HCC patients with varying clinical features (age, gender, stage, and grade) were analyzed using the Kaplan-Meier method according to the risk score.

We carried out a stratified analysis for further data mining (Figures 8(a)–8(h)). Following stratification by age, gender, tumor stage, and tumor grade, the risk score based on five CRGs signature performed as a significant prognostic indicator for age ≤ 65 (Figure 8(b)), male patients with HCC (Figure 8(d)), stages I-II (Figure 8(e)), grades 1-2 (Figure 8(g)), and grades 3-4 (Figure 8(h)).

### 3.8. Prognosis Model Validation in the ICGC Cohort

**Figure 9:**
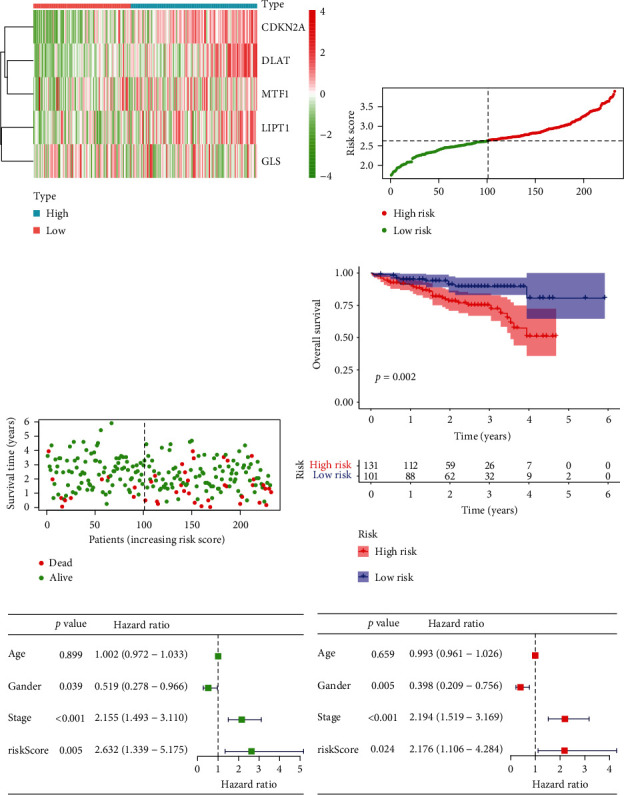
Verification of the five CRGs signature in the ICGC cohort. (a) Heatmaps of five prognostic CRGs in the ICGC database according to the risk score distribution. The color legend represents the log2 (FPKM) value. (b) The risk scores distribution. (c) The survival status of each patient is according to the risk score distribution. (d) Kaplan-Meier curves for the OS of HCC patients. (e, f) The independent survival analysis of the risk scores and clinical traits through univariate and multivariate Cox regression analysis.

We gathered comprehensive clinical information for 232 HCC cases from the ICGC database to serve as an external validation set. The risk score for each patient in the ICGC was computed according to the same formula created in the TCGA. The relationship between risk scores and clinical traits was examined (Supplementary Table 3). The TCGA cohort's median risk score was utilized to separate the ICGC cohort into high-risk and low-risk groups. 101 cases were located in the low-risk group, while the other 131 were in the high-risk group. The distribution diagram of risk scores and survival times displayed that the survival times of HCC patients in the ICGC decreased with rising risk scores, and in the low-risk group, there were more survivors than in the high-risk group (Figures 9(a)–9(c)). According to the Kaplan-Meier survival analysis, the survival time of high-risk patients was shorter than that of low-risk patients (*p* = 0.002, Figure 9(d)). Our risk model was also discovered to be an independent predictor of mortality in the ICGC (Figures 9(e) and 9(f)).

### 3.9. Prognostic Nomograms of HCC

**Figure 10:**
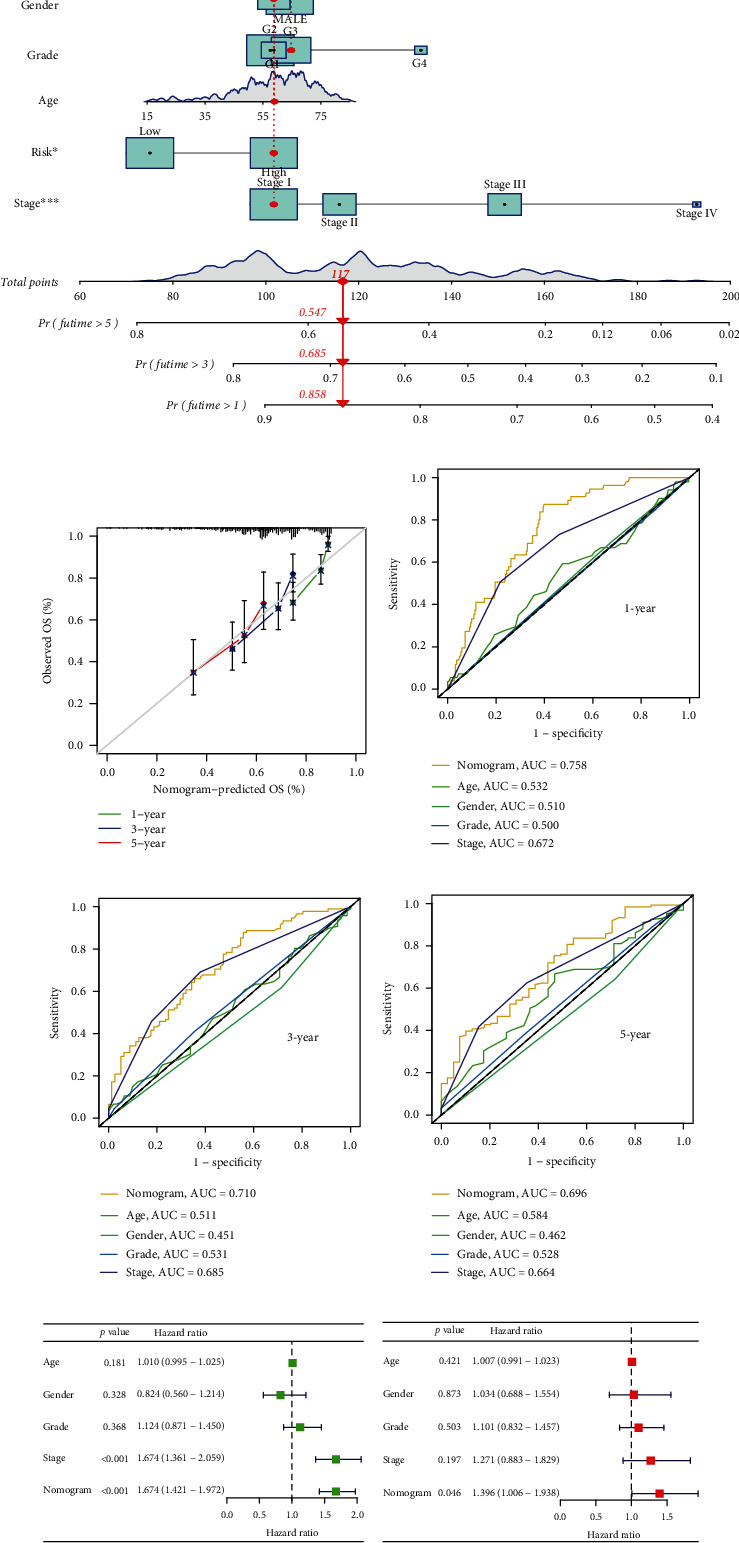
Predicting survival rates for HCC patients after one year, three years, and five years using the nomogram. (a) The nomogram model was formed to predict the survival rates of HCC patients in the TCGA cohorts. (b) Calibration curves of the nomogram. (c–e) The ROC curve explored the prognostic performance of the nomogram model. (f, g) Univariate and multivariate Cox analysis of the nomogram and clinical traits.

To further elevate the predictive power of our risk model, the nomograms were constructed by utilizing the five significant independent predictors (age, gender, grade, stage, and risk score) in the TCGA (Figure 10(a)). Good consistency between the prediction by nomogram and actual observation of 1-, 3-, and 5-year survival rates (Figure 10(b)) was confirmed by the calibration plot. The nomogram model also showed good prediction accuracy for the 1-, 3-, and 5-year OS rates. The relevant AUC values were 0.758, 0.710, and 0.696. (Figures 10(c)–10(e)). These findings suggest the preferable precision of the nomogram. In addition, the nomogram model could represent an independent risk factor in the TCGA (Figures 10(f) and 10(g)).

### 3.10. Function and Pathway Enrichment Analyses Based on Cuproptosis-Related Risk Score

**Figure 11:**
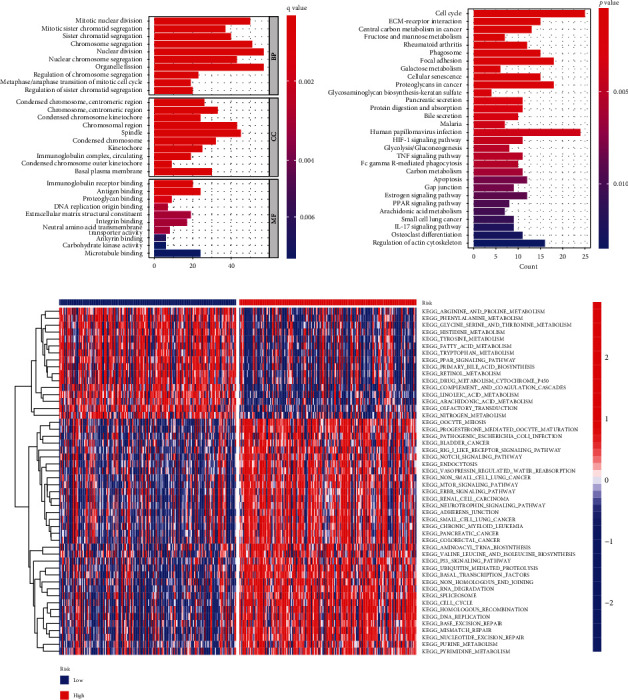
Functional enrichment analysis was performed according to the risk score. (a) A list of the top 10 significantly enriched GO terms. (b) A list of the top 30 most significantly enriched KEGG pathways. (c) The pathway activities scored by GSVA differently for high-risk and low-risk individuals.

For the assessment of the mechanisms underlying our risk model, we analyzed DEGs following the criteria: FDR < 0.05 and |log2FC| ≥ 1. 781 significant DEGs were identified, comprising 724 upregulated genes and 57 downregulated genes in the high-risk group. The GO terms were substantially enriched in chromosome segregation, nuclear division, chromosomal region, spindle, and DNA replication origin binding (Figure 11(a)). The majority of enriched KEGG pathways were cellular senescence, HIF-1 signaling pathway, TNF signaling pathway, apoptosis, cell cycle, and IL-7 signaling pathway (Figure 11(b)). As we could see, both GO terms and KEGG analysis indicated that the functional enrichment of the risk model highly correlated with cell cycle, cell death, and immune response. The results of GSVA revealed that low-risk group patients showed elevated expression levels of multiple metabolism pathways, like arginine and proline, phenylalanine, glycine, serine, and threonine, and high-risk group patients harbored upregulated expression levels of multiple cell cycle and tumorigeneses pathways, such as bladder cancer, notch signaling pathway, p53 signaling pathway, renal cell carcinoma, cell cycle, and DNA replication (Figure 11(c)).

### 3.11. Immune Characteristics Based on Cuproptosis-Related Risk Score

**Figure 12:**
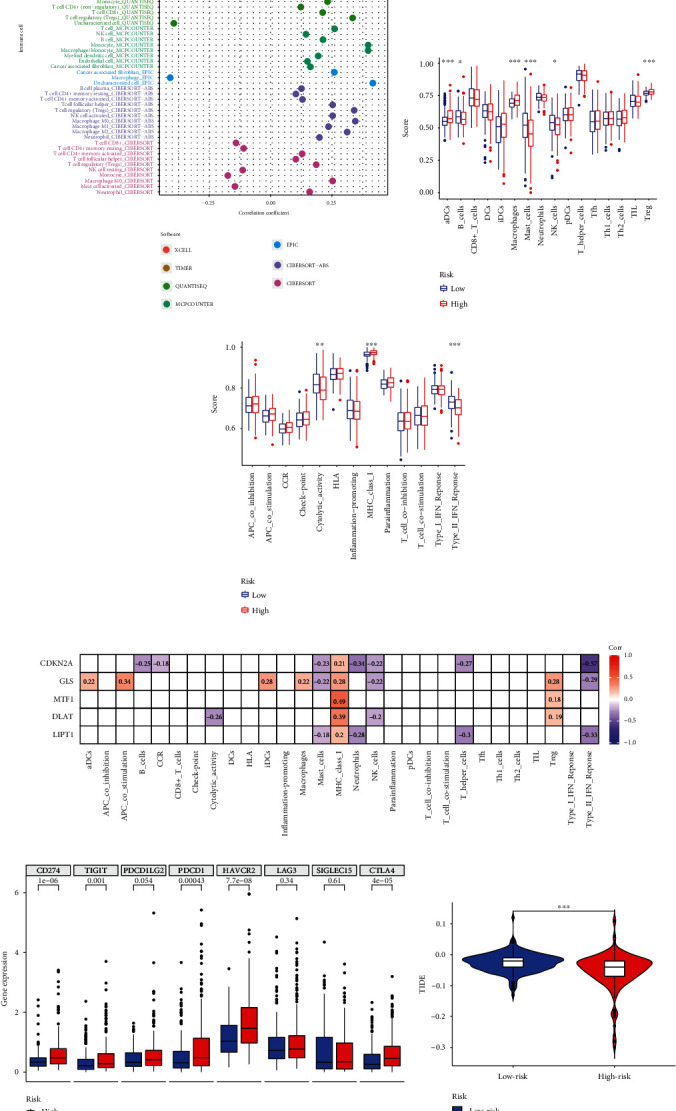
The immune landscape of cuproptosis-related risk score in HCC. (a) The forest plot displayed the connection between risk score and immune cell infiltration through TIMER, CIBERSORT, CIBERSORT-ABS, QUANTISEQ, MCPcounter, XCELL, and EPIC algorithms. (b, c) The bar graphs showed the difference in immune cell subpopulations and related functions between high-risk and low-risk groups. (d) The heatmap displayed the relationship of immune cell subpopulations and related functions with the five prognostic genes. (e) Differences in immune checkpoint expression between high-risk and low-risk groups. (f) The violin plots presented the TIDE scores between high-risk and low-risk groups. ^∗^*p* < 0.05, ^∗∗^*p* < 0.01, ^∗∗∗^*p* < 0.001.

To further explore the immune landscape of the cuproptosis-related risk model, we calculated the immune responses score through TIMER, CIBERSORT, CIBERSORT-ABS, QUANTISEQ, MCPcounter, XCELL, and EPIC algorithms. Then, the relationship between risk score and tumor immune response score was analyzed by Pearson's correlation. We formed a forest plot to display the detailed correlation coefficient between the immune cell infiltration and risk score (Figure 12(a)). Then, after we performed the ssGSEA method to explore the immune cell subpopulations and related functions, we found that immune cell subpopulations and related functions including aDCs, B cells, macrophages, mast cells, NK cells, Treg, cytolytic activity, MHC class I, and type II IFN response differed between high-risk and low-risk groups (Figures 12(b) and 12(c)). Besides, we further investigated the relationship between the five cuproptosis-related model genes and the ssGSEA result of immune cell subpopulations and related functions in each case (Figure 12(d)). We found some significantly positive correlations, such as the correlation between *MTF1* and MHC class I (*r* = 0.49), and some significantly negative correlations, such as the correlation between CDKN2A and type II IFN response (*r* = −0.57). As a crucial negative regulator of the tumor immune microenvironment, the immune checkpoints act as an essential role in assisting tumor cells in evading immune system attacks. Hence, we examined the expression level of eight important immune checkpoint genes. Between high-risk and low-risk groups, we discovered a significant variance in the expression level of immune checkpoints, including *CD274*, *TIGIT*, *PDCD1*, *HAVCR2*, and *CTLA4* (Figure 12(e)). Following that, a prediction of the immune checkpoint therapy response was made using the TIDE algorithm based on risk scores (Figure 12(f)). Interestingly, patients in the high-risk group had a higher likelihood of benefiting from immune checkpoint inhibitor therapy, suggesting that the risk score has the potential to predict whether HCC patients will benefit from immune checkpoint therapy.

## 4. Discussion

Copper overload can cause cuproptosis, which is a novel form of programmed cell death triggered by mitochondrial TCA cycles [12]. The relationship between tumors and copper has long been noted, and in fact, tumor tissue requires higher levels of copper [34]. Copper homeostasis imbalances can result in life-threatening conditions, such as Wilson's disease, in which most patients exhibit chronic liver disease with cirrhosis [35]. Copper overload also can lead to cirrhosis, which is one of the well-known risk factors for HCC [36]. Consequently, a better understanding of cuproptosis in HCC could be meaningful for developing new therapeutics. Here, through a series of analyses, we explored the relationship between cuproptosis and HCC. According to our findings, HCC has a different expression model of CRGs compared with normal liver tissue, and the different cuproptosis subtypes are strongly correlated with the clinical outcome of HCC patients. In addition, a prognostic risk model was created using different expressed CRGs. These findings may have implications for possible new therapeutic approaches to treating HCC.

Based on our study, we found that most of the CRGs are differentially expressed in HCC versus normal liver tissue. This finding is consistent with previous reports. Bian et al. found that most CRGs differ between clear cell renal cell carcinoma and normal renal cell [37]. Another report also indicated that most CRGs are differentially expressed in melanoma [38]. These clues suggested that the CRGs may have different expression patterns in tumors, including HCC, compared with normal tissues. Consensus clustering identified two clusters with significantly different OS based on the expressions of CRGs. We found the function, mutation, and immune analyses were performed differently between the two clusters, suggesting cuproptosis may be broadly related to HCC progression. The higher mutant frequency of *TP53* in cluster 1 and higher mutant frequency of *CTNNB1* in cluster 2 could help to elucidate the underlying molecular mechanism of the unique tumor microenvironment. HCC patients with *TP53* mutations have poorer outcomes [39], and the mutation status of *TP53* can be used to predict immune response to immunotherapy in a variety of cancer types [40, 41]. Thus, we showed that CRG expression might be closely related to HCC prognosis and tumor microenvironment.

We next constructed and validated an effective risk model with 5 CRGs (*MTF1*, *DLAT*, *GLS*, *CDKN2A*, *LIPT1*) for separating HCC patients into high-risk and low-risk groups. The model displayed good predictive ability in both the training and validation dataset. We also designed the nomogram to combine the CRGs risk score model and clinical features, and the nomogram showed excellent prediction with good calibration. All these five genes exhibited upregulated expression in HCC patients. As a classic metal sensing transcription factor, metal regulatory transcription factor 1 (*MTF1*) stimulates the expression of genes involved in metal homeostasis after exposure to heavy metals, including copper [42]. *MTF1* regulates hepatic *MT1/2* gene expression via a synergistic effect with *SIRT6*. By reducing ROS, inflammation, and tissue injury, *MT1/2* protects the liver from alcoholic liver disease [43]. Dihydrolipoamide S-acetyltransferase (*DLAT*) is one of the limited human proteins which can be lipoylated. Tsvetkov et al. discovered that lipoylated *DLAT* could bind copper and knocking out *DLAT* could prevent copper toxicity for cells [12]. *DLAT* encodes an essential subunit E2 of pyruvate dehydrogenase complex (*PDHC*), which is the critical autoantigen in primary biliary cholangitis (PBC) [44]. Cirrhosis and liver failure are associated with PBC [45]. In a recent study, it was found that posttranslational modifications of *PDHC* and *GLS* are involved in liver cancer metabolism and biogenesis [46]. There are two main types of *GLS*: kidney glutaminase (*GLS1*) and liver glutaminase (*GLS2*) [47]. The overexpression of *GLS2* in human liver cancer cells induced significant growth, proliferation, ectopic expression, and a G2/M arrest [48]. *CDKN2A* (also known as p16) is a tumor suppressor gene and one of the most frequently deleted genes in cancer genomes [49]. HCCs harboring deletions of *CDKN2A* constitute approximately 8% of cases [50, 51]. *CDKN2A* induces cell cycle arrest at G1 and G2 phases and inhibits the oncogenic effects of *CDK4/6* and *MDM2* [52]. In the TCA cycle, lipoyltransferase 1 (*LIPT1*) activates TCA cycle-associated 2-ketoacid dehydrogenases. The lack of LIPT1 can inhibit the TCA cycle [53]. There is little evidence that *LIPT1* is associated with tumor occurrence and development. Taken together, these five crucial genes, except *LIPT1*, contribute to the progression and development of liver disease or HCC.

Our study found that the phenotype of high-risk patients is more advanced, and the survival time is significantly shorter (Figures 6(g) and 6(h)). We hypothesized that cuproptosis resistance might be observed in high-risk patients, and cuproptosis might contribute to the poor outcomes of the patients in the high-risk group. *MTF1*, *GLS*, and *CDKN2A* were expressed at higher levels in these patients. Despite the two procuproptosis genes, *LIPT1* and *DLAT* were also upregulated in HCC patients. *LIPT1* is a key upstream regulator of protein lipoylation and a component of the lipoic acid pathway. *DLAT* is one of the protein targets of lipoylation [12]. Lipoylated *DLAT* could bind copper and take part in the regulation of cuproptosis. Thus, *LIPT1* and *DLAT* could regulate cuproptosis through posttranslational modifications, not only through the gene expression levels. Secondly, the high-risk patients were not enriched in fatty acid metabolism pathways. The high-risk group patients might therefore show resistance to cuproptosis due to suppressed related proteins of lipoylation.

Cuproptosis might inspire novel insights to treat tumors. Keeping intracellular copper levels within a specific range would be an effective treatment strategy for malignancies [54]. Copper ionophores, such as DSF and elesclomol, are emerging treatment options for cancers and exert their therapeutic effects by inducing cuproptosis. Many studies have demonstrated that, in combination with cupric ions, DSF may be beneficial for treating a variety of cancers in humans [14, 55, 56]. Elesclomol is particularly effective against tumors relying on mitochondrial metabolism [57]. The combination of elesclomol with paclitaxel has been well documented in clinical trials, particularly in advanced melanoma [58–60]. Overall, these findings suggest that copper ionophore-induced cuproptosis could be an effective therapeutic strategy for certain tumors. There is hope that HCC patients with low-risk scores might enjoy the antitumor impact of the copper ionophores. Additionally, we found significant differences in the expression levels of the typical immune checkpoint and TIDE score between the high-risk and low-risk groups, suggesting our risk model may also show good predictability of response to ICB.

Our study has some weaknesses. First, the results may be biased since the small number of patients. Second, although this prognostic model demonstrated robust predictive ability in both TCGA and ICGC databases, there is no clinical data to further validate it, which is urgently warranted in future research. Third, these critical genes of this model require more experiments *in vitro* and *in vivo* to verify, which is underway in our laboratory.

## 5. Conclusions

In conclusion, CRGs were significantly differentially expressed between HCC and normal liver tissues, and the prognosis of HCC patients is significantly influenced by cuproptosis. A novel prognostic model containing five CRGs has been conducted for HCC prognosis prediction. High-risk HCC patients had a poor prognosis, advanced disease stages, and an enhanced therapeutic response. These results may shed light on new molecular pathways involved in HCC carcinogenesis and enable the prediction of treatment outcomes for HCC patients. Additional *in vitro* and *in vivo* studies to validate these results would be beneficial.

## Data Availability

The RNA-seq and clinical data used to support the discoveries of this analysis were collected from the TCGA (https://portal.gdc.cancer.gov/) and ICGC (https://dcc.icgc.org/) databases.

## Supplementary Materials

Supplementary Materials Description of the three supplementary tables. Supplementary Table 1: immune cell infiltration between the two clusters via various methods. Supplementary Table 2: the relationship between the risk score of cuproptosis and clinicopathologic features in the TCGA database. Supplementary Table 3: the relationship between the risk score of cuproptosis and clinicopathologic features in the ICGC database.
