# Supplementary material for: Prognostic and Immunological Significance of the Molecular Subtypes and Risk Signatures Based on Cuproptosis in Hepatocellular Carcinoma
Source: Mediators Inflamm. 2023 Apr 20;2023:3951940. doi: 10.1155/2023/3951940 (PMC10139815; doi:10.1155/2023/3951940)
Supplement: Supplementary Materials — Description of the three supplementary tables. Supplementary Table 1: immune cell infiltration between the two clusters via various methods. Supplementary Table 2: the relationship between the risk score of cuproptosis and clinicopathologic features in the TCGA database. Supplementary Table 3: the relationship between the risk score of cuproptosis and clinicopathologic features in the ICGC database. [file 3951940.f1.zip › Supplementary Table 1.docx]

Supplementary Table 1. Immune cell infiltration between the two clusters via various methods.

| Methods | Immune cell | *p* |
| --- | --- | --- |
| TIMER | B cell_TIMER | 0.000389 |
| TIMER | T cell CD4+_TIMER | 0.001105 |
| TIMER | Neutrophil_TIMER | 1.58E-08 |
| TIMER | Macrophage_TIMER | 5.21E-08 |
| TIMER | Myeloid dendritic cell_TIMER | 6.20E-06 |
| CIBERSORT | B cell naive_CIBERSORT | 0.006592 |
| CIBERSORT | T cell CD4+ memory activated_CIBERSORT | 0.01952 |
| CIBERSORT | T cell follicular helper_CIBERSORT | 0.004287 |
| CIBERSORT | T cell regulatory (Tregs)_CIBERSORT | 0.000176 |
| CIBERSORT | Monocyte_CIBERSORT | 0.002731 |
| CIBERSORT | Macrophage M0_CIBERSORT | 0.009787 |
| CIBERSORT | Mast cell activated_CIBERSORT | 0.001434 |
| CIBERSORT-ABS | T cell CD4+ memory activated_CIBERSORT-ABS | 0.020255 |
| CIBERSORT-ABS | T cell follicular helper_CIBERSORT-ABS | 2.68E-06 |
| CIBERSORT-ABS | T cell regulatory (Tregs)_CIBERSORT-ABS | 1.01E-07 |
| CIBERSORT-ABS | NK cell activated_CIBERSORT-ABS | 0.0009 |
| CIBERSORT-ABS | Macrophage M0_CIBERSORT-ABS | 0.000117 |
| CIBERSORT-ABS | Macrophage M1_CIBERSORT-ABS | 0.010329 |
| CIBERSORT-ABS | Macrophage M2_CIBERSORT-ABS | 0.000413 |
| CIBERSORT-ABS | Mast cell activated_CIBERSORT-ABS | 0.044743 |
| CIBERSORT-ABS | Neutrophil_CIBERSORT-ABS | 0.04236 |
| QUANTISEQ | B cell_QUANTISEQ | 0.007099 |
| QUANTISEQ | Macrophage M1_QUANTISEQ | 9.66E-06 |
| QUANTISEQ | Macrophage M2_QUANTISEQ | 0.014467 |
| QUANTISEQ | Monocyte_QUANTISEQ | 2.67E-05 |
| QUANTISEQ | NK cell_QUANTISEQ | 0.015589 |
| QUANTISEQ | T cell CD4+ (non-regulatory)_QUANTISEQ | 5.37E-05 |
| QUANTISEQ | T cell CD8+_QUANTISEQ | 8.79E-06 |
| QUANTISEQ | T cell regulatory (Tregs)_QUANTISEQ | 1.07E-05 |
| QUANTISEQ | uncharacterized cell_QUANTISEQ | 5.67E-06 |
| MCPCOUNTER | T cell_MCPCOUNTER | 3.28E-07 |
| MCPCOUNTER | T cell CD8+_MCPCOUNTER | 0.014804 |
| MCPCOUNTER | B cell_MCPCOUNTER | 0.00259 |
| MCPCOUNTER | Monocyte_MCPCOUNTER | 0.000134 |
| MCPCOUNTER | Macrophage/Monocyte_MCPCOUNTER | 0.000134 |
| XCELL | B cell_XCELL | 5.78E-06 |
| XCELL | T cell CD4+ memory_XCELL | 1.44E-05 |
| XCELL | T cell CD4+ central memory_XCELL | 0.04741 |
| XCELL | T cell CD8+ naive_XCELL | 0.007085 |
| XCELL | Class-switched memory B cell_XCELL | 0.025831 |
| XCELL | Common lymphoid progenitor_XCELL | 2.89E-10 |
| XCELL | Endothelial cell_XCELL | 1.31E-13 |
| XCELL | Granulocyte-monocyte progenitor_XCELL | 0.001841 |
| XCELL | Hematopoietic stem cell_XCELL | 2.48E-11 |
| XCELL | Macrophage_XCELL | 0.047305 |
| XCELL | Macrophage M2_XCELL | 0.000107 |
| XCELL | T cell NK_XCELL | 1.07E-05 |
| XCELL | Plasmacytoid dendritic cell_XCELL | 0.034209 |
| XCELL | T cell gamma delta_XCELL | 0.002334 |
| XCELL | T cell CD4+ Th1_XCELL | 0.003181 |
| XCELL | T cell CD4+ Th2_XCELL | 1.55E-16 |
| XCELL | stroma score_XCELL | 1.17E-11 |
| XCELL | microenvironment score_XCELL | 0.00072 |
| EPIC | Cancer associated fibroblast_EPIC | 0.029637 |
| EPIC | Macrophage_EPIC | 4.03E-06 |
| EPIC | uncharacterized cell_EPIC | 1.80E-06 |
