# Supplementary material for: Prognostic and Immunological Significance of the Molecular Subtypes and Risk Signatures Based on Cuproptosis in Hepatocellular Carcinoma
Source: Mediators Inflamm. 2023 Apr 20;2023:3951940. doi: 10.1155/2023/3951940 (PMC10139815; doi:10.1155/2023/3951940)
Supplement: Supplementary Materials — Description of the three supplementary tables. Supplementary Table 1: immune cell infiltration between the two clusters via various methods. Supplementary Table 2: the relationship between the risk score of cuproptosis and clinicopathologic features in the TCGA database. Supplementary Table 3: the relationship between the risk score of cuproptosis and clinicopathologic features in the ICGC database. [file 3951940.f1.zip › Supplementary Table 2.docx]

| Characteristic | Low risk | High risk | *p* |
| --- | --- | --- | --- |
| n | 185 | 185 |  |
| Gender, n (%) |  |  | 0.375 |
| Female | 65 (17.6%) | 56 (15.1%) |  |
| Male | 120 (32.4%) | 129 (34.9%) |  |
| Grade, n (%) |  |  | < 0.001 |
| G1 | 18 (4.9%) | 37 (10.1%) |  |
| G2 | 80 (21.9%) | 97 (26.6%) |  |
| G3 | 77 (21.1%) | 44 (12.1%) |  |
| G4 | 8 (2.2%) | 4 (1.1%) |  |
| Stage, n (%) |  |  | < 0.001 |
| Stage I | 66 (19.1%) | 105 (30.3%) |  |
| Stage II | 50 (14.5%) | 35 (10.1%) |  |
| Stage III | 53 (15.3%) | 32 (9.2%) |  |
| Stage IV | 3 (0.9%) | 2 (0.6%) |  |
| T, n (%) |  |  | < 0.001 |
| T1 | 71 (19.3%) | 110 (30%) |  |
| T2 | 56 (15.3%) | 37 (10.1%) |  |
| T3 | 48 (13.1%) | 32 (8.7%) |  |
| T4 | 10 (2.7%) | 3 (0.8%) |  |
| M, n (%) |  |  | 0.622 |
| M0 | 132 (48.9%) | 134 (49.6%) |  |
| M1 | 3 (1.1%) | 1 (0.4%) |  |
| N, n (%) |  |  | 0.623 |
| N0 | 129 (50.4%) | 123 (48%) |  |
| N1 | 3 (1.2%) | 1 (0.4%) |  |
| Age, median (IQR) | 62 (52, 68) | 61 (51, 69) | 0.592 |

Supplementary Table 2. The relationship between the risk score of cuproptosis and clinicopathologic features in the TCGA database.
