# Supplementary material for: Prognostic and Immunological Significance of the Molecular Subtypes and Risk Signatures Based on Cuproptosis in Hepatocellular Carcinoma
Source: Mediators Inflamm. 2023 Apr 20;2023:3951940. doi: 10.1155/2023/3951940 (PMC10139815; doi:10.1155/2023/3951940)
Supplement: Supplementary Materials — Description of the three supplementary tables. Supplementary Table 1: immune cell infiltration between the two clusters via various methods. Supplementary Table 2: the relationship between the risk score of cuproptosis and clinicopathologic features in the TCGA database. Supplementary Table 3: the relationship between the risk score of cuproptosis and clinicopathologic features in the ICGC database. [file 3951940.f1.zip › Supplementary Table 3.docx]

Supplementary Table 3. The relationship between the risk score of cuproptosis and clinicopathologic features in the ICGC database.

| Characteristic | Low risk | High risk | *p* |
| --- | --- | --- | --- |
| n | 131 | 101 |  |
| Gander, n (%) |  |  | 0.536 |
| Female | 37 (15.9%) | 24 (10.3%) |  |
| Male | 94 (40.5%) | 77 (33.2%) |  |
| Stage, n (%) |  |  | 0.109 |
| Stage I | 14 (6%) | 22 (9.5%) |  |
| Stage II | 63 (27.2%) | 43 (18.5%) |  |
| Stage III | 41 (17.7%) | 30 (12.9%) |  |
| Stage IV | 13 (5.6%) | 6 (2.6%) |  |
| Age, median (IQR) | 69 (63, 74) | 67 (58, 74) | 0.083 |
